# Supplementary material for: Symmetry regimes for circular photocurrents in monolayer MoSe2
Source: Nat Commun. 2018 Aug 21;9:3346. doi: 10.1038/s41467-018-05734-z (PMC6104061; doi:10.1038/s41467-018-05734-z)
Supplement: Supplementary file 1 — Supplementary Information [file 41467_2018_5734_MOESM1_ESM.pdf]

Supplementary information to:
Symmetry regimes for circular photocurrents in monolayer $MoSe_2$

Jorge Quereda[1]*, Talieh S. Ghiasi[1], Jhih-Shih You[2], Jeroen van den Brink[2], Bart J. van Wees[1], Caspar H. van der Wal[1]

[1] Zernike institute for Advanced Materials, University of Groningen, NL-9747AG Groningen, The Netherlands

[2] Institute for Theoretical Solid State Physics, IFW Dresden, Helmholtzstr. 20, 01069 Dresden, Germany

CONTENT

1. AFM characterization

2. Optical microscopy images of the fabrication process and the final device

3. Electrical characterization of the 1L-$MoSe_2$ phototransistors

4. Color map of the CPC amplitude as a function of V_{ds} and V_{gate} for illumination at normal incidence

5. Comparison of the photovoltage and photocurrent measurements and consistency checks

6. Theoretical analysis of the photogalvanic and photon drag effects

7. Spectral characterization of CPC amplitude for different voltages.

8. Brief note on valley exciton transitions in monolayer $MoSe_2$

Supplementary Note 1: AFM characterization

We measure the height profile of the BN-encapsulated $MoSe_2$ on a SiO_2/Si substrate by AFM. The thickness of both of the $MoSe_2$ and the top h-BN flakes are measured as 0.7 nm (Supplementary Figure 1b) which corresponds to monolayer $MoSe_2$ and bilayer h-BN, in agreement with the reported values in literature[1,2]. The bottom h-BN has a thickness of 7.65 nm (21-22 layers). The AFM images also reveal the presence of bubbles due to trapped molecules in the h-BN/$MoSe_2$ interface. Reportedly, the accumulation of the interface contaminants in these bubbles ensures a perfectly clean interface at the bubble-free regions, and is a signature of the good adhesion between the two layers.[3]

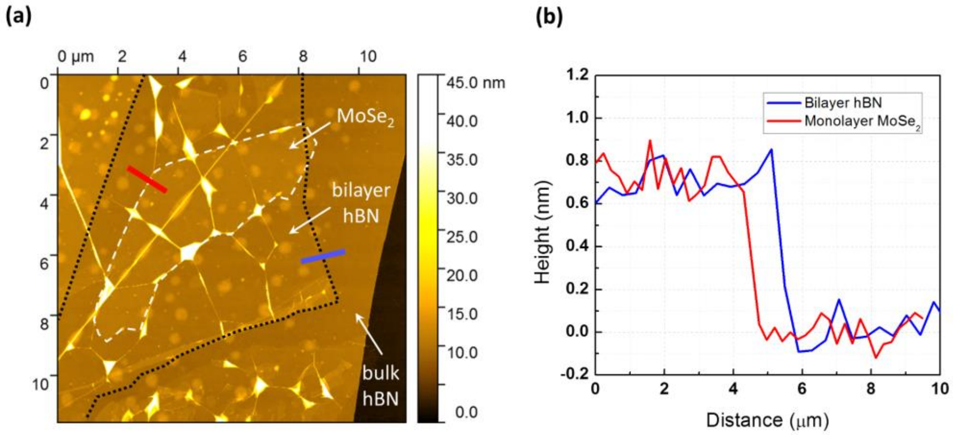

Supplementary Figure 1 (a) AFM image of the BN-encapsulated $MoSe_2$ on SiO_2/Si substrate. The dashed lines highlight the edge of the flakes. (b) Height profile along the red and blue lines indicated in panel (a), corresponding to the edges of the monolayer $MoSe_2$ and the bilayer h-BN flakes. For clarity, The profiles are offsetted to the same zero level.

Supplementary Note 2: Optical microscopy images of the fabrication process and the final device

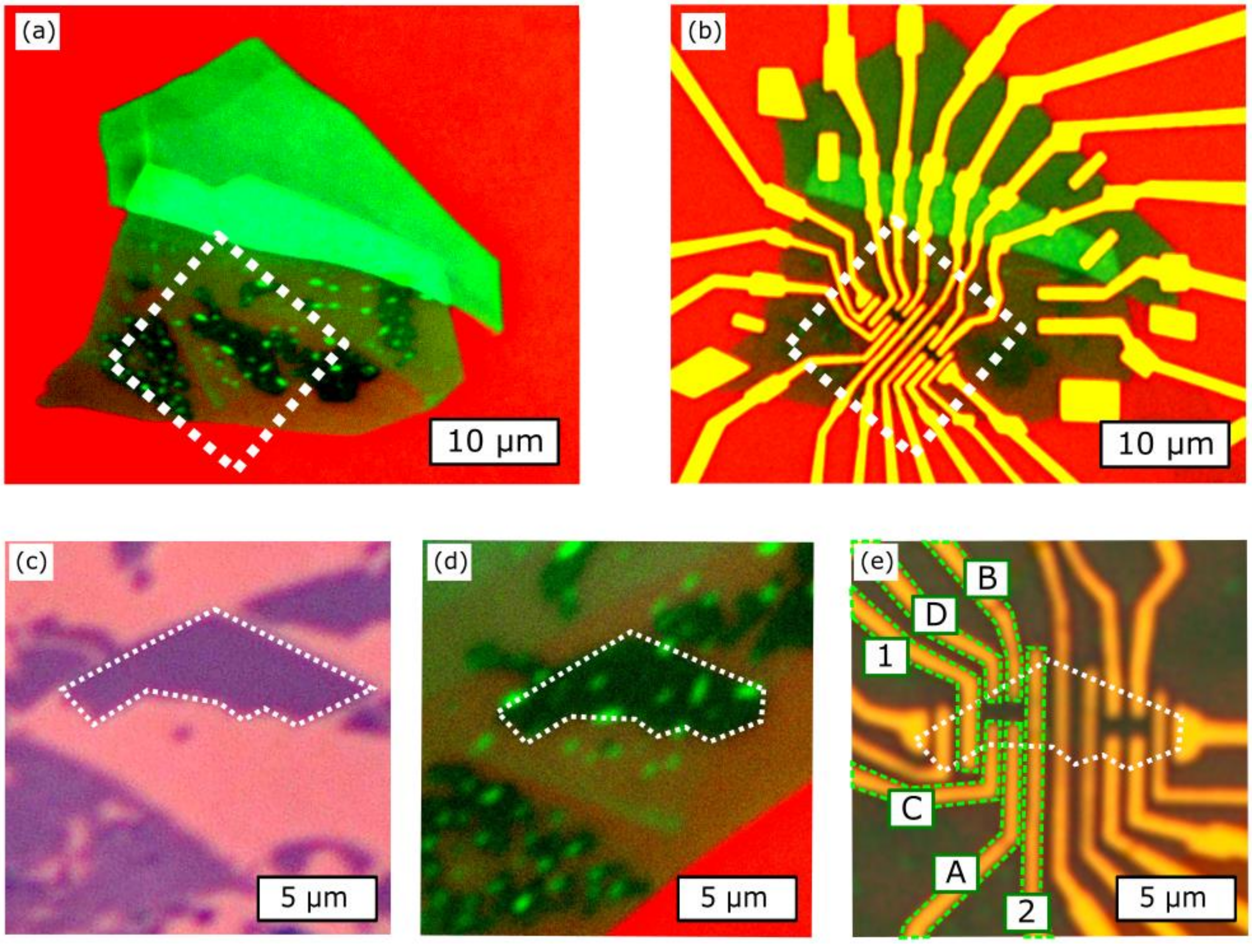

Supplementary Figure 2 Enhanced-contrast optical images of the device fabrication process (a) Fully encapsulated 1L-$MoSe_2$ crystal before the fabrication of the contacts. (b) Final device. (c-e) Zoom in of the region indicated by a dotted square in panels (a) and (b) at the different stages of the fabrication process. (c) Exfoliated 1L-$MoSe_2$ flake on SiO_2 before processing. (d) Same flake shown in (c) after encapsulation with top bilayer h-BN and bottom multilayer h-BN. (e) Final device with the fabricated contacts on top of the BN/MoS_2/BN stack. The electrodes used for the measurements of the main text are highlighted in green. The dashed white line indicates the edges of the 1L-$MoSe_2$ flake.

Supplementary Note 3: Electrical characterization of the 1L-$MoSe_2$ phototransistors

The DC electrical characterization of the sample is performed in the dark while keeping the sample in vacuum (10^{-4} mbar). In order to obtain the electrical transport properties of the $MoSe_2$ channel, we perform four-terminal measurements in Hall-bar geometry. We apply a source-drain current on the contacts 1 and 2 (See figure 1a in the main text) and measure the voltage drop along the channel using the Hall contacts A and C. We remark that using the contacts that only partially cover the channel is preferable for the characterization of the intrinsic electrical properties of the $MoSe_2$ channel, since this allows to prevent the formation of depletion regions near the metal contacts.[4]

Supplementary Figure 3a shows a transfer characteristic for the 1L-$MoSe_2$ phototransistor, presenting a clear n-type behavior. We extract the threshold gate voltage (V_{th}) of 19 V as the gate voltage at which the conductivity starts to increase. We estimate a field-effect mobility of about 17 cm^2/V.s from the linear fit to the transfer curve, for the range of gate voltage ($V_{gate} > V_{th}$) with linear dependence of conductivity. Supplementary Figure 3b shows the four-terminal I-V characteristics of the phototransistor. The ohmic response of the channel can be readily observed from the linearity of the obtained I-Vs. The inset in Supplementary Figure 3b shows the square resistance of the $MoSe_2$ channel, R_{sq}, obtained as the slope of the linear fit to the I-V divided by the length-to-width ratio of the $MoSe_2$ channel, as a function of the gate voltage.

In our device geometry, encapsulation of the $MoSe_2$ channel with h-BN reduces the influence of the adsorbate molecules on the $MoSe_2$ surface and prevents charge scatterings due to interface impurities and the Si substrate, which largely reduces the hysteresis in the charge transport measurements. Moreover, the bilayer h-BN plays the role of a tunnel barrier for injection of charge carriers, preventing the level pinning at the contacts.

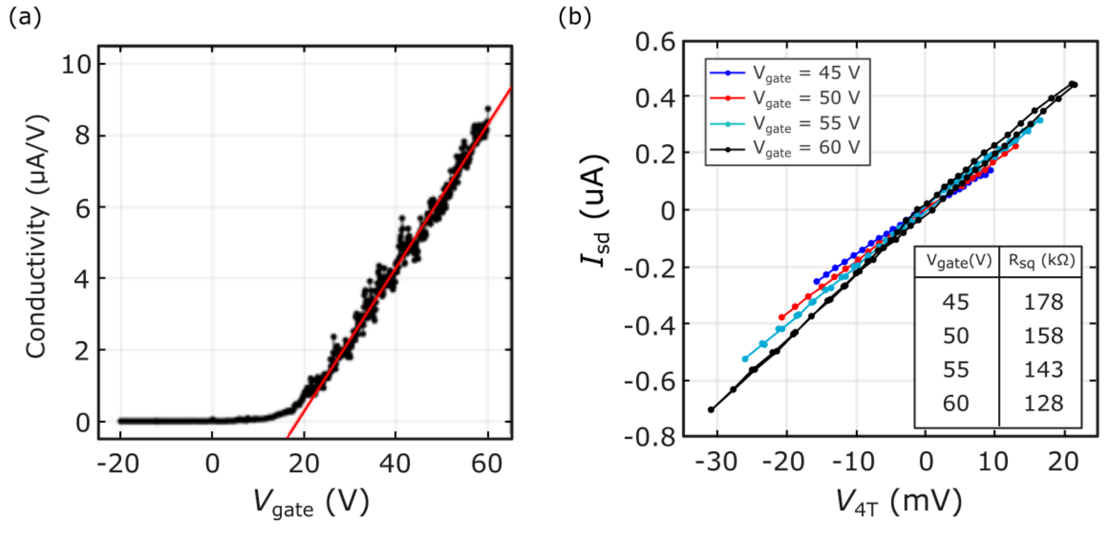

Supplementary Figure 3 Electrical characterization of the channel in 4-terminal geometry. (a) Channel conductivity as a function of gate voltage. The red line is a linear fit to the data for $V_{gate} > V_{th.}$ (b) I-V characteristics of the channel and the square resistances, estimated for the gate voltages of 40 to 60 V (shown in the legend).

Supplementary Note 4: Color map of the CPC amplitude as a function of V_{ds} and V_{gate} for illumination at normal incidence.

Supplementary Figure 4 shows a colormap of the CPC amplitude C as a function of the drain-source and gate voltages for $\phi = 0^{o}$. The value of C remains near zero regardless of the applied voltages. This allows us to rule out that the dominant contribution to our observed CPC signals is a Berry phase-induced CPGE, since it should become maximal for normal incidence. This measurement also rules out that our signals have a significant contribution from the valley-Hall effect, since such effect would appear as a nonzero contribution to the CPC linear with the drain-source voltage. The absence of the valley-Hall effect in our device can be understood since this effect has been reported for studies on highly n-doped devices, and it increases with the gate voltage. In our device, the 1L-$MoSe_2$ channel only starts to open for $V_{gate} > 20$ V. Thus, a much larger doping could be required for observing the valley-Hall effect.

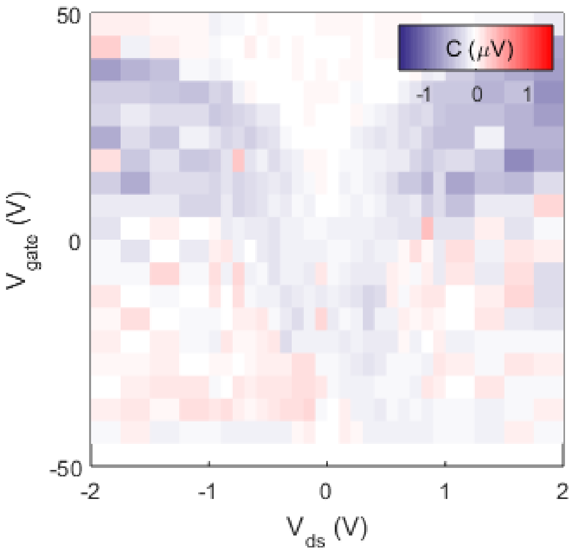

Supplementary Figure 4 Colormap of the CPC amplitude, C as a function of the drain-source and gate voltages, V_{ds} and V_{gate} for normal incidence angle, $\phi = 0$ degrees.

Supplementary Note 5: Comparison of the photovoltage and photocurrent measurements and consistency checks

Supplementary Figure 5 shows the helicity-dependent open-circuit photovoltage and short-circuit photocurrent measured in two sets of electrodes: [A, B] and [1, 2]. These results are representative for a wider range of checks that we performed, where we always found a linear relation between the observed values for C, L_1 and L_2 in the current and voltage signals. This photoresponse can thus be measured equivalently as current or voltage signals on our device. In addition, for C we observed no dependence on the orientation of the linear polarization for the laser beam incident on the $\lambda/4$ plate.

Finally, Figs. 2b and 3c (main text) show that the spectral dependence of C is preserved for two different sets of electrodes, further ruling out a role for specific contacts or standing-wave effects between electrodes. Our full range of consistency checks allows us to rule out that effects at specific electrodes, and effects from confining light between the micron-scale metallic electrode structure, give a significant contribution to the helicity-dependent signals that we analyze.

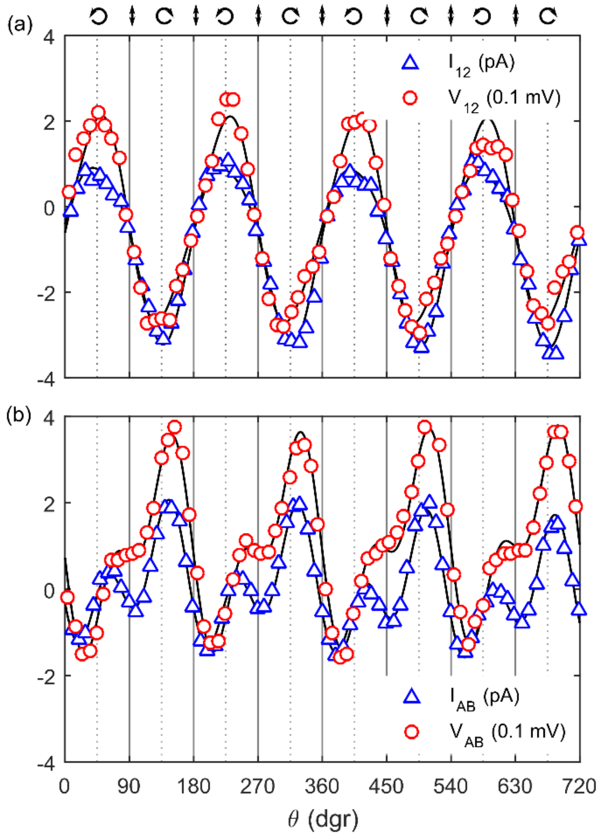

Supplementary Figure 5 Helicity-dependent open-circuit photovoltage (red circles) and short-circuit photocurrent (blue circles) for electrodes [1, 2] (a) and [A, B] (b) at $V_{gate} = 0$ V and $V_{ds} = 0$, as a function of the waveplate angle (θ). The black solid lines are fittings to the phenomenological equation (1) in the main text. Except for a scale factor, the θ dependence of the photovoltage and photocurrent are very similar, as expected from the linear I-V of the semiconductor channel.

Supplementary Figure 6 shows the power dependence of C, L_1 and L_2 for electrodes A and B and measuring photocurrent instead of photovoltage, in contrast with the measurements shown in the main text for contacts 1 and 2 (Figure 1c). This shows that the same linear power dependence appears regardless of the contacts or the measurement technique.

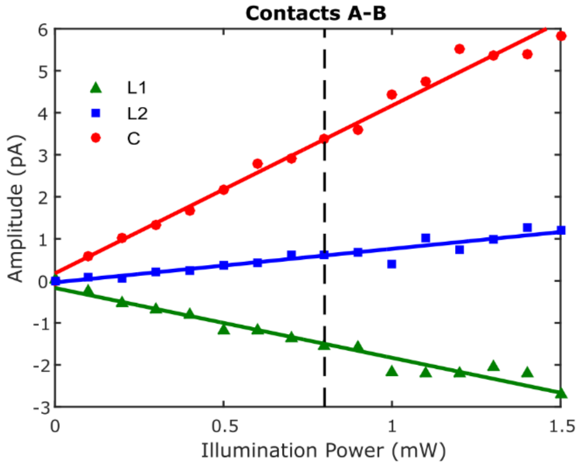

Supplementary Figure 6 Illumination power dependence of L_1, L_2 and C extracted from the fittings of helicity-dependent photocurrent measurements (as those shown in Supplementary Figure 5) for contacts A and B.

Supplementary Note 6: Theoretical analysis of the photogalvanic and photon drag effects

I. General description of photogalvanic and photon drag effects

The theoretical foundations of the photogalvanic and photon drag effects (PGE and PDE) trace back to several decades ago.[5–7] More specifically, they are characterized by a DC current generated by a time-varying electric field, with amplitude proportional to the square of the applied field. This current is generated by photoelectrons which are excited by optical (vertical in the band structure) transitions and, depending on its microscopic origin, can depend on the polarization (linear PCE and PDE) or the helicity (circular PGE and PDE) of the applied field. Recent studies on derivations of PGE and PDE rely on the nonlinear susceptibility [8], Floquet theory [9] and the kinetic equation approach.

Let us consider the situation of a 2D material illuminated by a monochromatic light source, with complex electric field defined as the plane wave

$$E_j(\vec{r},t) = E_j e^{i\omega t + i\vec{q}\cdot\vec{r}} + E_j^* e^{i\omega t - i\vec{q}\cdot\vec{r}}, \tag{1}$$

where the subindices *i, j* and *k* stand for the Cartesian coordinates, ω is the angular frequency and the wave vector $\vec{q}$ can be expressed in spherical coordinates as

$$\vec{q} = -q(\sin(\phi)\cos(\alpha), \sin(\phi)\sin(\alpha), \cos(\phi)). \tag{2}$$

We assume that the incident light forms a polar angle ϕ and an azimuthal angle α with the 2D plane (see Figure 1 in the main text). We can write down the electric field (as well as the vector potential $\vec{A} = \vec{E}/i\omega$) as

$$\vec{E} = \begin{pmatrix} E_x \\ E_y \\ E_z \end{pmatrix} = E_0 \begin{pmatrix} -i\sin(2\theta)\sin(\alpha) + (1 - i\cos(2\theta))\cos(\phi)\cos(\alpha) \\ i\, sin(2\theta)\cos(\alpha) + (1 - i\cos(2\theta))\cos(\phi)\sin(\alpha) \\ -(1 - i\cos(2\theta))\sin(\phi) \end{pmatrix}, \tag{3}$$

with E_0 as the magnitude of the applied electric field and θ as the angle between the fast axis of the $\lambda/4$ waveplate and the initial linear polarization of the light. $\theta = \pi/4$, $(3\pi/4)$ for left (right) circularly polarized light. For future purpose, here we write $(\vec{E} \times \vec{E}^*)$ as

$$\begin{aligned} \left(\vec{E} \times \vec{E}^*\right)_x &= -2i\cos(\alpha)\sin(2\theta)\sin(\phi)\,E_0^2 \\ \left(\vec{E} \times \vec{E}^*\right)_y &= -2i\sin(\alpha)\sin(2\theta)\sin(\phi)\,E_0^2 \\ \left(\vec{E} \times \vec{E}^*\right)_z &= -2i\sin(2\theta)\cos(\phi)\,E_0^2 \end{aligned} \tag{4}$$

The light-induced current density $\vec{J}$ inside the material can be generically written in series of the Cartesian components (*l, j, k*) of the electric field $\vec{E}$. Per component of $\vec{J}$ this gives

$$J_l = \sigma_{lj} E_j e^{-i\omega t + i\vec{q}\vec{r}} + \sigma_{ljk}^{(2')} E_j E_k e^{-2i\omega t + 2i\vec{q}\vec{r}} + \sigma_{ljk}^{(2)} E_j E_k^* + \cdots, \tag{5}$$

where σ_{lj} is a second rank tensor and $\sigma_{ljk}^{(2)}$, and $\sigma_{ljk}^{(2')}$ are third rank tensors. The first and second terms in the right correspond respectively to a linear AC current (at optical frequency) in response to the electric field and an AC

current of twice the frequency of the radiation, responsible for second harmonic generation. The relevant term for us is the third term, which corresponds to a DC current $J_{\mathrm{l}}^{\mathrm{DC}}$ in response to the oscillating field:

$$J_{\mathrm{l}}^{\mathrm{DC}} = \sigma_{ljk}^{(2)} E_j E_k^* \ , \tag{6}$$

By doing a Taylor expansion over the wave vector q we can rewrite J_l^{DC} as

$$J_l^{\mathrm{DC}} = \sigma_{ljk}^{(2)}(\omega, \vec{q}) E_j E_k^* = \chi_{ljk}(\omega) E_j E_k^* + T_{ljk\mu}(\omega) q_\mu E_j E_k^* + \cdots \tag{7}$$

Here $\chi_{ljk} = \sigma_{ljk}^{(2)}(\omega, 0)$ does not depend on the radiation wave vector $\vec{q}$ and is responsible for the photogalvanic effect, $\vec{J}^{\mathrm{PGE}}$, while $T_{ljk\mu}$ accounts for the photon drag effect $\vec{J}^{\mathrm{PDE}}$, linear with factors q_l.

Requirement of inversion symmetry breaking

We now show that the absence of inversion symmetry is necessary for getting nonzero photogalvanic and photon drag effect. First, we note that J_l^{DC} is antisymmetric (changes its sign) under inversion of the spatial coordinates *x, y, z* → *-x, -y, -z*, while the object $E_j E_k^*$ is symmetric under that transformation. In consequence, if the inversion transformation is a symmetry of the studied system, J_l^{DC} cannot have any dependence on $E_j E_k^*$ and χ_{ljk} must be zero. In other words, the photogalvanic effect can only emerge in systems with broken inversion symmetry.

Linear and circular photogalvanic and photon drag effect

Next, we observe that, since the current density must be real, it cannot change under complex conjugation. In consequence, from equation (7) we get $\chi_{lkj}^* = \chi_{ljk}$. Therefore, the real part of χ_{ljk} is symmetric under coordinate exchange while its imaginary part is antisymmetric under this operation. This allows us to rewrite the photogalvanic current as follows,

$$J_l^{\mathrm{PGE}} = \chi_{ljk} E_\beta E_\gamma^* = \chi_{ljk}^{\mathrm{sym}} E_j E_k^* + i \chi_{ljk}^{\mathrm{antisym}} E_j E_k^* \ , \tag{8}$$

or, using the transformation under permutation of the subindices *j* and *k*,

$$J_l^{\mathrm{PGE}} = \frac{1}{2} \chi_{ljk}^{\mathrm{sym}} \left(E_j E_k^* + E_k E_j^* \right) + \frac{1}{2} \chi_{ljk}^{\mathrm{antisym}} \left(E_j E_k^* - E_k E_j^* \right) \equiv J_l^{\mathrm{LPGE}} + J_l^{\mathrm{CPGE}} \tag{9}$$

We can now compare J_l^{LPGE} and J_l^{CPGE} with the Stokes parameters:

$$S_1 = \frac{|E_x|^2 - |E_y|^2}{|E_x|^2 + |E_y|^2}; \qquad S_2 = \frac{E_x E_y^* + E_x^* E_y}{|E_x|^2 + |E_y|^2}; \qquad S_3 = i \frac{E_x E_y^* - E_x^* E_y}{|E_x|^2 + |E_y|^2} \tag{10}$$

We see that J_l^{LPGE} is proportional to S_2, which accounts for the linearly polarized radiation (linear photogalvanic effect), while J_l^{CPGE} is proportional to S_3, and, therefore, it is sensitive to the circularly polarized radiation (circular photogalvanic effect).

Finally, the totally antisymmetric Levi-Civita tensor ϵ_{sjk} can be used to contract $\chi_{ljk}^{\text{antisym}}$ to only one pseudo vector index,

$$\sum_{jk} \chi_{ljk}^{\text{antisym}} \left(E_j E_k^* - E_k E_j^*\right) = i \sum_{sjk} 2\gamma_{ls} \epsilon_{sjk} \left(E_j E_k^* - E_k E_j^*\right) = i \sum_{s} \gamma_{ls} \left(\vec{E} \times \vec{E}^*\right)_s \ , \tag{11}$$

where γ_{ls} is a second rank pseudo-tensor and *l* and *s* stand for Cartesian coordinates. Thus J_l^{CPGE} can be expressed as [5]

$$J_l^{\text{CPGE}} = i \sum_{j} \gamma_{lj} \left(\vec{E} \times \vec{E}^*\right)_j \ , \tag{12}$$

It is convenient to separate J_l^{PDE} in a similar fashion into its circular and linear polarization sensitive components, J_l^{CPDE} and J_l^{LPDE}. For J_l^{CPDE} we get:

$$J_l^{\text{CPDE}} = \ i \sum_{jk} T_{ljk} q_j \left(\vec{E} \times \vec{E}^*\right)_k \ , \tag{13}$$

At this point it is worth noting that we have not still made any assumption about the physical origin of J_l^{CPGE}. Thus, equations (12) and (13) are completely general and must hold regardless of the underlying physical mechanism.

II. Symmetry arguments for the CPC in monolayer TMDCs.

In the following, we use symmetry arguments to determine the nonzero components of the tensors γ_{ij} and T_{lsj}, as defined in equations (12) and (13). This allows to extract constraints for the dependence of J_l^{CPGE} and J_l^{CPDE} on the illumination angles α and ϕ. We remark again that, since equations (12) and (13) must hold regardless of the physical origin of the CPC, the discussion below is completely general.

In order of decreasing symmetry we analyze three cases: D_{3h}, C_{3v}, and *Single mirror-plane* symmetry. We find that crystal structures belonging to the high-symmetry class D_{3h} cannot support any CPGE. For the case of C_{3v} symmetry with an oblique incidence angle ϕ, only γ_{xy} can have a nonzero value, which then gives a nonzero CPGE, but always with the property that it flips signs upon reversal of ϕ (in conflict with a BC origin). Systems with only one mirror symmetry can not only have nonzero γ_{xy} but also nonzero γ_{yz} and γ_{xz}, allowing for BC-CPGE. We conclude that our experimental results for low source-drain voltage are only compatible with, at most, one mirror-plane symmetry, since otherwise γ_{xz} and γ_{yz} cancel out and, therefore, the photocurrent cannot be preserved upon inversion of the incidence angle, ϕ.

D_{3h} symmetry

The scenario of a 1L-TMDC system with ideal mirror symmetry with respect to the crystal plane (symmetric environments and external fields) give the system the high D_{3h} symmetry. This has a three-fold rotation symmetry around the z axis, defined by the operator C_3, three two-fold axes perpendicular to C_3, a mirror plane in the xy plane, defined by $\sigma_h = \begin{pmatrix} 1 & 0 & 0 \\ 0 & 1 & 0 \\ 0 & 0 & -1 \end{pmatrix}$ and three mirror vertical planes with respect to the xy plane σ_v. The improper rotation is $\sigma_h C_3$.

In the section directly below here on C_{3v} symmetry we derive that the CPGE current is

$$\vec{j}^{CPGE} = i \begin{pmatrix} 0 & \gamma_{xy} & 0 \\ -\gamma_{xy} & 0 & 0 \\ 0 & 0 & 0 \end{pmatrix} \begin{pmatrix} (\vec{E} \times \vec{E}^*)_x \\ (\vec{E} \times \vec{E}^*)_y \\ (\vec{E} \times \vec{E}^*)_z \end{pmatrix} = i \begin{pmatrix} \gamma_{xy}(\vec{E} \times \vec{E}^*)_y \\ -\gamma_{xy}(\vec{E} \times \vec{E}^*)_x \\ 0 \end{pmatrix}. \tag{14}$$

This result for C_{3v} can be extended to the case for D_{3h} by adding the requirement for the additional mirror symmetry σ_h. This brings that a pseudo-vector $(\vec{E} \times \vec{E}^*)$ becomes $-\sigma_h(\vec{E} \times \vec{E}^*)$, and gives the condition $\gamma_{xy} = 0$. Consequently, all CPGE current contributions cancels out for the D_{3h} symmetry.

For CPDE, the C_{3v} symmetry (below) yields

$$\vec{j}^{CPDE} = i(T_{yxz} + T_{yzx})q_z \begin{pmatrix} -(\vec{E} \times \vec{E}^*)_y \\ (\vec{E} \times \vec{E}^*)_x \\ 0 \end{pmatrix} \propto \begin{pmatrix} -\sin(\alpha)\sin(2\theta)\sin(2\phi) \\ \cos(\alpha)\sin(2\theta)\sin(2\phi) \\ 0 \end{pmatrix}. \tag{15}$$

A direct extension of this analysis shows that the additional mirror plane σ_h does not impose further constrains on $\vec{j}^{CPDE}$. Thus, CPDE photocurrents can be present for this symmetry.

C_{3v} symmetry

If we assume a 1L-TMDC crystal symmetry in the plane, but drop the assumption of mirror symmetry with respect to the crystal plane (relevant, for example, for a 1L-TMDC sustained on a substrate), the system has C_{3v} symmetry. This corresponds to a three-fold rotation symmetry around the z axis C_3, and three mirror planes perpendicular to the xy plane. The CPGE photocurrent is given by

$$j_i^{CPGE} = i\,\gamma_{ij}(\vec{E} \times \vec{E}^*)_j. \tag{16}$$

Under a 2π/3 rotation

$$R = \begin{pmatrix} \cos(2\pi/3) & \sin(2\pi/3) & 0 \\ -\sin(2\pi/3) & \cos(2\pi/3) & 0 \\ 0 & 0 & 1 \end{pmatrix},$$

$\vec{E} \times \vec{E}^*$ becomes $R(\vec{E} \times \vec{E}^*)$ and $\vec{j}^{CPGE}$ becomes $R\vec{j}^{CPGE}$. Since γ should remain the same under the rotational symmetry, we have

$$R\vec{j}^{CPGE} = i\gamma R\left(\vec{E} \times \vec{E}^*\right) . \tag{17}$$

By replacing (26) into (27) we obtain

$$R\gamma = \gamma R . \tag{18}$$

An additional constraint is given by the mirror symmetry. There are three mirror planes perpendicular to the *xy* plane, and we assume that the angle between the mirror plane and the *x* axis is ψ. Under the mirror reflection, characterized by the operator $= \begin{pmatrix} \cos(2\psi) & \sin(2\psi) & 0 \\ \sin(2\psi) & -\cos(2\psi) & 0 \\ 0 & 0 & 1 \end{pmatrix}$, γ should remain the same, while $\vec{j}^{CPGE}$ becomes $M\vec{j}^{CPGE}$. As a pseudo-vector, $\left(\vec{E} \times \vec{E}^*\right)$ becomes $-M\left(\vec{E} \times \vec{E}^*\right)$. Therefore, we obtain

$$M\gamma = -\gamma M . \tag{19}$$

Combining the constraints from rotational and mirror symmetries, we conclude that γ_{ij} has only one independent parameter:

$$\gamma_{ij} = \begin{pmatrix} 0 & \gamma_{xy} & 0 \\ -\gamma_{xy} & 0 & 0 \\ 0 & 0 & 0 \end{pmatrix}, \tag{20}$$

and the CPGE current must have a form of

$$\vec{j}^{CPGE} = i\begin{pmatrix} 0 & \gamma_{xy} & 0 \\ -\gamma_{xy} & 0 & 0 \\ 0 & 0 & 0 \end{pmatrix}\begin{pmatrix} \left(\vec{E} \times \vec{E}^*\right)_x \\ \left(\vec{E} \times \vec{E}^*\right)_y \\ \left(\vec{E} \times \vec{E}^*\right)_z \end{pmatrix} \propto \gamma_{xy}\begin{pmatrix} \sin(\alpha)\sin(2\theta)\sin(\phi) \\ -\cos(\alpha)\sin(2\theta)\sin(\phi) \\ 0 \end{pmatrix}. \tag{21}$$

This description yields a nonzero CPGE current that indeed shows a $\sin(2\theta)$ dependence in the polarization control. Further, it only yields nonzero CPGE currents for nonzero angles ϕ. For this symmetry group the CPGE current changes sign when the incidence angle is switched from ϕ to $-\phi$.

For the CPDE, we have $J_l^{\mathrm{CPDE}} = iT_{ljk}q_j\left(\vec{E} \times \vec{E}^*\right)_k$. Imposing that, for a transverse electromagnetic wave, the vector $\left(\vec{E} \times \vec{E}^*\right)$ should be along the same direction as the photon momentum $\vec{q}$, we get $q_j\left(\vec{E} \times \vec{E}^*\right)_k = q_k\left(\vec{E} \times \vec{E}^*\right)_j$. Imposing the invariance of $\vec{j}^{CPDE}$ under a rotation of $2\pi / 3$, $\vec{j}^{CPDE\prime} = R\vec{j}^{CPDE}$ we get

$$T_{zij}R_{ik}q_kR_{jl}\left(\vec{E} \times \vec{E}^*\right)_l = T_{zij}q_i\left(\vec{E} \times \vec{E}^*\right)_j . \tag{22}$$

From each $q_i\left(\vec{E} \times \vec{E}^*\right)_j$ we get

$$T_{zxz} + T_{zzx} = T_{zyz} + T_{zzy} = T_{zxy} + T_{zyx} = 0 ;$$
$$T_{zyy} = T_{zxx} . \tag{23}$$

Also, from j_x and j_y we get

$$T_{xzz} = 0\,;\; T_{xyy} = -T_{xxx}\,;\; T_{yxx} = \frac{\sqrt{3}}{2}T_{xxx}\,;\; T_{yxy} + T_{yyx} = -2T_{xxx}\,;$$
$$T_{yxz} + T_{yzx} = -\left(T_{xyz} + T_{xzy}\right);\; T_{yyz} + T_{yzy} = T_{xxz} + T_{xzx}\,; T_{yyy} = -\tfrac{1}{2}\left(T_{xxy} + T_{xyx}\right). \tag{24}$$

Combining this with the restrictions imposed by the mirror symmetry we find that only $T_{yxz} + T_{yzx} = -\left(T_{xyz} + T_{xzy}\right) \equiv \chi$ remains and

$$\vec{J}^{CPDE} = i\left(T_{yxz} + T_{yzx}\right)q_z\begin{pmatrix} -\left(\vec{E}\times\vec{E}^*\right)_y \\ \left(\vec{E}\times\vec{E}^*\right)_x \\ 0 \end{pmatrix} \propto \begin{pmatrix} -\sin(\alpha)\sin(2\theta)\sin(2\phi) \\ \cos(\alpha)\sin(2\theta)\sin(2\phi) \\ 0 \end{pmatrix}. \tag{25}$$

Remarkably, under C_{3v} symmetry we find that a CPGE must have a sin(ϕ) dependence, while a CPDE must have a sin(2ϕ) dependence. They can thus be distinguished by their dependence on ϕ.

Single mirror-plane symmetry

Now we consider a case of even lower symmetry: that of a 1L-TMDC system that has just one mirror plane which is perpendicular to the *xy* plane. In a real device, the presence of asymmetric electrodes and strain gradients is expected to lead to this low-symmetry situation (or even lower symmetry). In particular, this scenario is relevant for strained monolayer $MoSe_2$, since the lowest stiffness for deformation occurs along the armchair direction in the crystal. We cannot assume that this crystal direction has a known relation with the *xy* coordinate frame (defined by the experimental geometry with electrodes, see Fig. 1 main text). We therefore introduce the angle ψ to describe angle between the mirror plane and the *x* axis.

Under the mirror reflection, $\vec{J}^{CPGE}$ becomes $M\vec{J}^{CPGE}$, where $M = \begin{pmatrix} \cos(2\psi) & \sin(2\psi) & 0 \\ \sin(2\psi) & -\cos(2\psi) & 0 \\ 0 & 0 & 1 \end{pmatrix}$. Once more, as a pseudovector, $\left(\vec{E}\times\vec{E}^*\right)$ becomes $-M\left(\vec{E}\times\vec{E}^*\right)$. Therefore, we obtain $M\gamma = -\gamma M$. The absence of the 2π/3 rotational symmetry in this case allows more independent parameters to appear in γ_{ij} and the CPGE current takes the form

$$\vec{J}^{CPGE} = i\begin{pmatrix} 0 & \gamma_{xy} & \gamma_{xz} \\ -\gamma_{xy} & 0 & \gamma_{yz} \\ \gamma_{zx} & \gamma_{zy} & 0 \end{pmatrix}\begin{pmatrix} \left(\vec{E}\times\vec{E}^*\right)_x \\ \left(\vec{E}\times\vec{E}^*\right)_y \\ \left(\vec{E}\times\vec{E}^*\right)_z \end{pmatrix} \propto \begin{pmatrix} [\gamma_{xy}(\sin(\alpha)\sin(\phi) + \gamma_{xz}\cos(\phi)]\sin(2\theta) \\ [-\gamma_{xy}\left(\cos(\alpha)\sin(\phi) + \gamma_{yz}\cos(\phi)\right]\sin(2\theta) \\ 0 \end{pmatrix}, \tag{26}$$

where we assumed that the j_z^{CPGE} component must be zero for a single-layer crystal.

The mirror symmetry yields the additional conditions:

$$\gamma_{xz} = \frac{1-\cos(2\psi)}{\sin(2\psi)}\gamma_{yz} \quad \text{and} \tag{27}$$

$$\gamma_{zx} = \frac{1-\cos(2\psi)}{\sin(2\psi)}\gamma_{zy}\;. \tag{28}$$

Thus, we get a CPGE contribution independent of α and changing as $\cos(\phi)$. In the next section we will show that this angular dependence is required for a Berry curvature-induced CPGE.

Now we consider the CPDE. For simplicity, we assume that the mirror symmetry is from x to $-x$. We get

$$\begin{aligned} j_x^{CPDE} &= 2\sin(2\theta)\Big\{T_{xzz}\cos^2(\phi) + \frac{T_{xyz}+T_{xzy}}{2}\sin(\alpha)\sin(2\phi) \\ &\quad + \left(T_{xxx}\cos^2(\alpha) + T_{xyy}\sin^2(\phi)\right)\sin^2(\phi)\Big\} \ ; \\ j_y^{CPDE} &= \sin(2\theta)\left\{\left(T_{yxz}+T_{yzx}\right)\cos(\alpha)\sin(2\phi) + \left(T_{yxy}+T_{yyx}\right)\sin(2\alpha)\sin^2(\phi)\right\} \ ; \\ j_z^{CPDE} &= \sin(2\theta)\left\{\left(T_{zxz}+T_{zzx}\right)\cos(\alpha)\sin(2\phi) + \left(T_{zxy}+T_{zyx}\right)\sin(2\alpha)\sin^2(\phi)\right\} ; \end{aligned} \tag{29}$$

Here, we find that CPDE can have a contribution dependent on $\sin(2\alpha)\sin^2(\phi)$, which matches the observed angular dependence for the low-V_{ds} regime.

As discussed in the main text, this symmetry analysis does not bring forward the $\sin(3\alpha)$ dependence experimentally observed for the large-V_{ds} regime (although this could still come forward from asymmetric transport properties of the zig-zag and armchair directions of the $MoSe_2$ crystal).

In the particular case where an additional mirror symmetry in the z direction is allowed (original D_{3h} case subject to e.g. uniaxial strain), the CPGE and CPDE current is further constrained, yielding

$$\vec{j}^{CPGE} = i\begin{pmatrix} 0 & 0 & \gamma_{xz} \\ 0 & 0 & \gamma_{yz} \\ \gamma_{zx} & \gamma_{zy} & 0 \end{pmatrix}\begin{pmatrix} \left(\vec{E}\times\vec{E}^*\right)_x \\ \left(\vec{E}\times\vec{E}^*\right)_y \\ \left(\vec{E}\times\vec{E}^*\right)_z \end{pmatrix} \propto \begin{pmatrix} \gamma_{xz}\cos(\phi)\sin(2\theta) \\ \gamma_{yz}\cos(\phi)\sin(2\theta) \\ 0 \end{pmatrix}, \tag{30}$$

$$\vec{j}^{CPDE} = \begin{pmatrix} (T_{xyz}+T_{xzy})\sin(\alpha)\sin(2\phi)\sin(2\theta) \\ (T_{yxz}+T_{yzx})\cos(\alpha)\sin(2\phi)\sin(2\theta) \\ (T_{zyz}+T_{zzx})\cos(\alpha)\sin(2\phi)\sin(2\theta) \end{pmatrix}, \tag{31}$$

and only the $\cos(\phi)$-dependent CPGE contribution can still appear. Also, for the CPDE current, the terms on $\sin(2\alpha)\sin^2(\phi)$ fade out. Thus, we see that our experimental results require a broken out-of-plane mirror symmetry.

III. Berry curvature and circular photogalvanic effect

In this section, we show how a circular photogalvanic current can emerge as a consequence of a nonzero Berry curvature. We start the derivation of the photocurrent equation based on the assumption that the momentum of light is small and can be ignored. Thus, we consider only vertical optical interband transition between the initial and final bands. The photocurrent $\vec{J}$ can be derived based on the Fermi-Golden rule [10]:

$$J_{\mathrm{i}} = -\frac{2\pi e\tau}{\hbar}\sum_{I,F}\frac{d^2k}{(2\pi)^2}f_{IF}(\vec{k})\left(v_F^i - v_I^i\right)\delta(\Delta E_{FI}-\omega)|D|^2 \tag{32}$$

where v is the group velocity of the electron state, ω is the excitation energy, $\Delta E_{FI} = E_F(\vec{k}) - E_I(\vec{k})$ and $f_{IF}(\vec{k}) \equiv f_I(\vec{k}) - f_F(\vec{k})$ are the differences of energy and equilibrium Fermi distribution function between the initial and final states. $|I\rangle$ and $|F\rangle$ are the Bloch wavefunction of the initial and final states. D is the optical transition dipole defined as

$$D = \frac{e}{m_e} \langle F| \vec{A} \cdot \vec{p} \, |I\rangle \ , \tag{33}$$

where e and m_e are the charge and mass of a bare electron, $\vec{A}$ is the vector potential of light and $\vec{p}$ is the momentum operator defined as $\vec{p} = (m_e/i\hbar)[\vec{r}, H]$. Here we assume τ is the relaxation time for all bands.

Now we consider the light as

$$E = \begin{pmatrix} E_x \\ E_y \\ E_z \end{pmatrix} = \begin{pmatrix} -i\sin(2\theta)\sin(\alpha) + (1 - i\cos(2\theta))\cos(\phi)\cos(\alpha) \\ i\sin(2\theta)\sin(\alpha) + (1 - i\cos(2\theta))\cos(\phi)\cos(\alpha) \\ -(1 - i\,cos(2\theta))\sin(\phi) \end{pmatrix}, \tag{34}$$

with E_0 as the magnitude of the electric field and $\vec{A} = \vec{E}/i\omega$ Therefore under this polarized light $|D^2|$ is given as

$$|D|^2 = \left(\frac{\omega e}{m_e}\right)^2 \left|\langle F|E_x p_x + E_y p_y + E_z p_z|I\rangle\right|^2. \tag{35}$$

We further rewrite $E_a E_b^*$ (the subindices refer to Cartesian coordinates) as

$$E_a E_b^* = \{E_a E_b^*\} + [E_a E_b^*] \ , \tag{36}$$

where $\epsilon_{sab}[E_a E_b^*] = \frac{1}{2}\left(\vec{E} \times \vec{E}^*\right)_s$ with

$$\left(\vec{E} \times \vec{E}^*\right)_x = -2i\cos(\alpha)\sin(2\theta)\sin(\phi)E_0^2 \ ; \tag{37}$$

$$\left(\vec{E} \times \vec{E}^*\right)_y = -2i\sin(\alpha)\sin(2\theta)\sin(\phi)E_0^2 \ ; \tag{38}$$

$$\left(\vec{E} \times \vec{E}^*\right)_z = -2i\sin(2\theta)\cos(\phi)E_0^2 \, . \tag{39}$$

In the following, we drop $\{E_a E_b^*\}$ and $|E_a|^2$ terms which do not contribute to the circular photogalvanic effect, characterized by the $\sin(2\theta)$ dependence. Therefore, we obtain

$$\begin{aligned} |D|^2 = \frac{1}{2}\left(\frac{\omega e}{m_e}\right)^2 \Big[&\left(\vec{E} \times \vec{E}^*\right)_z \left(\langle F|p_x|I\rangle\langle I|p_y|F\rangle - \langle F|p_y|I\rangle\langle I|p_x|F\rangle\right) \\ &-\left(\vec{E} \times \vec{E}^*\right)_y \left(\langle F|p_x|I\rangle\langle I|p_z|F\rangle - \langle F|p_z|I\rangle\langle I|p_x|F\rangle\right) \\ &+\left(\vec{E} \times \vec{E}^*\right)_x \left(\langle F|p_y|I\rangle\langle I|p_z|F\rangle - \langle F|p_z|I\rangle\langle I|p_y|F\rangle\right)\Big] . \end{aligned} \tag{40}$$

Here, the $\sin(2\theta)$ factor that governs the currents changes sign when the helicity of light is inverted.

In a 2D crystal, we can use Peierls substitution, $p_i = \frac{m_e}{\hbar}\left[z, \hat{H}\right]$ for $i = x, y$. Because translational symmetry is broken along the *z* direction for a 2D crystal, we use $p_z = im_e/\hbar\left[z, \hat{H}\right]$ in $|D|^2$,

$$|D|^2 = \left(\frac{\omega \mathrm{E}_0 e}{\hbar}\right)^2 \Bigg[-i\cos(\phi)\sin(2\theta)\left(\langle F|\frac{\partial \hat{H}}{\partial k_x}|I\rangle\langle I|\frac{\partial \hat{H}}{\partial k_y}|F\rangle - \langle F|\frac{\partial \hat{H}}{\partial k_y}|I\rangle\langle I|\frac{\partial \hat{H}}{\partial k_x}|F\rangle\right)$$
$$+\sin(\alpha)\sin(\phi)\sin(2\theta)\left(\langle F|\frac{\partial \hat{H}}{\partial k_x}|I\rangle\langle I|\left[z,\hat{H}\right]|F\rangle - \langle F|\left[z,\hat{H}\right]|I\rangle\langle I|\frac{\partial \hat{H}}{\partial k_x}|F\rangle\right) \quad (41)$$
$$-\cos(\alpha)\sin(\phi)\sin(2\theta)\left(\langle F|\frac{\partial \hat{H}}{\partial k_y}|I\rangle\langle I|\left[z,\hat{H}\right]|F\rangle - \langle F|\left[z,\hat{H}\right]|I\rangle\langle I|\frac{\partial \hat{H}}{\partial k_y}|F\rangle\right)\Bigg].$$

The first term in $|D|^2$ can be related to Berry curvature (BC) for the electronic Bloch states of the nth band:

$$\Omega_n^z(\vec{k}) = i\hat{z}\cdot\left(\nabla_{\vec{k}} u_{n\vec{k}}^*\right)\times\left(\nabla_{\vec{k}} u_{n\vec{k}}\right) = -2\sum_{n\neq n'} \frac{\mathrm{Im}\left(\langle u_{n\vec{k}}|\frac{\partial \hat{H}}{\partial k_x}|u_{n'\vec{k}}\rangle\langle u_{n'\vec{k}}|\frac{\partial \hat{H}}{\partial k_y}|u_{n\vec{k}}\rangle\right)}{\left[E_n(\vec{k}) - E_{n'}(\vec{k})\right]^2}. \quad (42)$$

In a 2D crystal the Berry curvature has only a nonzero component, perpendicular to the *xy* plane (the Berry curvature behaves as a pseudoscalar). In a N-band system, the BC of the nth band comes from all the other N - 1 bands. Therefore the photocurrent Eq. (32) needs to sum over all possible initial and final states that satisfy the energy conservation $\delta(\Delta E_{\mathrm{FI}} - \omega)$. For a simple two-band approximation, *F* stands for the conduction band (CB) and *I* for the valance band (VB) with the definition of Berry curvature,

$$\Omega_F^z(\vec{k}) = -\Omega_I^z(\vec{k}) = \frac{2\,\mathrm{Im}\left(\langle CB|\frac{\partial \hat{H}}{\partial k_x}|VB\rangle\langle VB|\frac{\partial \hat{H}}{\partial k_y}|CB\rangle\right)}{\left[E_{CB}(\vec{k}) - E_{VB}(\vec{k})\right]^2}. \quad (43)$$

This approximation allows us to simplify $|D|^2$ as

$$|D|^2 = \left(\frac{\Omega \mathrm{E}_0 e}{\hbar}\right)^2 \Bigg[-i\cos(\phi)\sin(2\theta)\,\Omega_F^z(\vec{k})(\Delta E_{FI})^2$$
$$+\sin(\alpha)\sin(\phi)\sin(2\theta)\left(\langle F|\frac{\partial \hat{H}}{\partial k_x}|I\rangle\langle I|z|F\rangle - \langle F|z|I\rangle\langle I|\frac{\partial \hat{H}}{\partial k_x}|F\rangle\right)\Delta E_{FI} \quad (44)$$
$$-\cos(\alpha)\sin(\phi)\sin(2\theta)\left(\langle F|\frac{\partial \hat{H}}{\partial k_y}|I\rangle\langle I|z|F\rangle - \langle F|z|I\rangle\langle I|\frac{\partial \hat{H}}{\partial k_y}|F\rangle\right)\Delta E_{FI}\Bigg].$$

For multi-band cases, $\Omega_{\mathrm{I}}^z(\vec{k}) \neq \Omega_{\mathrm{F}}^z(\vec{k})$ and we could have additional corrections from other bands to the Berry curvature [4] of the forms

$$-2\sum_{n\neq F,I}\frac{\mathrm{Im}\left(\langle u_{n\vec{k}}|\frac{\partial\hat{H}}{\partial k_x}|u_{I\vec{k}}\rangle\langle u_{I\vec{k}}|\frac{\partial\hat{H}}{\partial k_y}|u_{n\vec{k}}\rangle\right)}{\left[E_n(\vec{k})-E_I(\vec{k})\right]^2} \tag{45}$$

In pristine monolayer TMDCs, the conduction and valence band edges can be well-described by a two-band (without the inclusion of spins) or four-band (with the inclusion of spins), massive Dirac fermion model (see ref. [11] and, for a recent review, ref. [12]). In this effective model (supported by first principle calculations), the Berry curvatures at the conduction and valence bands take opposite values, $\Omega_{\mathrm{F}}^{z}\,(k) = -\Omega_{\mathrm{I}}^{z}\,(k)$, where F stands for the conduction bands (CB) and I for the valence band (VB). The Berry curvature is dependent on spin through the spin-dependent band gap. In this description, the Fermi golden rule with two-band approximation allows us to connect the photocurrent to the Berry curvature with no additional corrections. TMDCs with lower symmetry are well described by a tilted Dirac model[13] and the Berry curvature is also found to take opposite values for conduction and valence band edges in this case. However, first principle calculations are needed for understanding the higher band corrections to the photocurrent.

One could also consider a possible role for intraband transitions, as discussed in ref. [14]. However, these intraband contributions can only occur for $\hbar\omega < 2\mu$, where $\hbar\omega$ is the the photon energy and μ is the chemical potential (choosing $\mu = 0$ at the bottom of the conduction band). In our measurements, the photon energy is around 1.6 eV, well above the chemical potential, in the order of 0.1-0.2 eV even when a gate voltage is applied. Thus , such effects are not expected to give a strong contribution here, but could still be observed in infrared to microwave regime.

In equation (44) the first term of $|D^2|$ is from Berry curvature $\Omega_F^z(\vec{k})$ and shows that this contribution to the CPGE is independent of α, and maximal for normal incidence, $\phi = 0$. As discussed in the main text and in Supplementary Section 4, we do not find any contribution to CPC that satisfies this angular dependence. It is worth noting that, from the general definition of J_1^{CPGE}, equation (12), we find that a CPGE contribution changing as $\cos(\phi)\,\sin(2\theta)$ is associated with the matrix elements γ_{xz} and γ_{yz}. The symmetry arguments discussed above confirm that these matrix elements can only be nonzero if the device symmetry is reduced to, at most, a single mirror plane. Therefore, the D_{3h} symmetry of 1L-$MoSe_2$ must be reduced (for example from device asymmetries or strain gradients) in order to allow for a Berry curvature-induced CPGE (BC-CPGE).

Supplementary Note 7: Spectral characterization of CPC amplitude for different voltages

Supplementary Figure 7 shows the CPC amplitude C as a function of the illumination wavelength for different combinations of voltages, V_{ds} and V_g. The resonant character of C is clearly observed for all measurements, with the maximum signal occurring at 785 nm for $V_{ds} = 1$ V and at 790 nm for $V_{ds} = 0$ V (see also Fig. 3c in the main text).

For the data acquired at $V_{ds} = 1$ V, a weaker but nonzero CPC is also observed for off-resonance excitation with energies above the 1L-$MoSe_2$ absorption edge, which could be associated to the emergence of free-electron driven CPC. At negative gate voltages, this CPC contribution even presents a different sign from that of the main peak at ~785 nm. Although relevant, a comprehensive analysis of this off-resonance CPC contribution is beyond the scope of our present work.

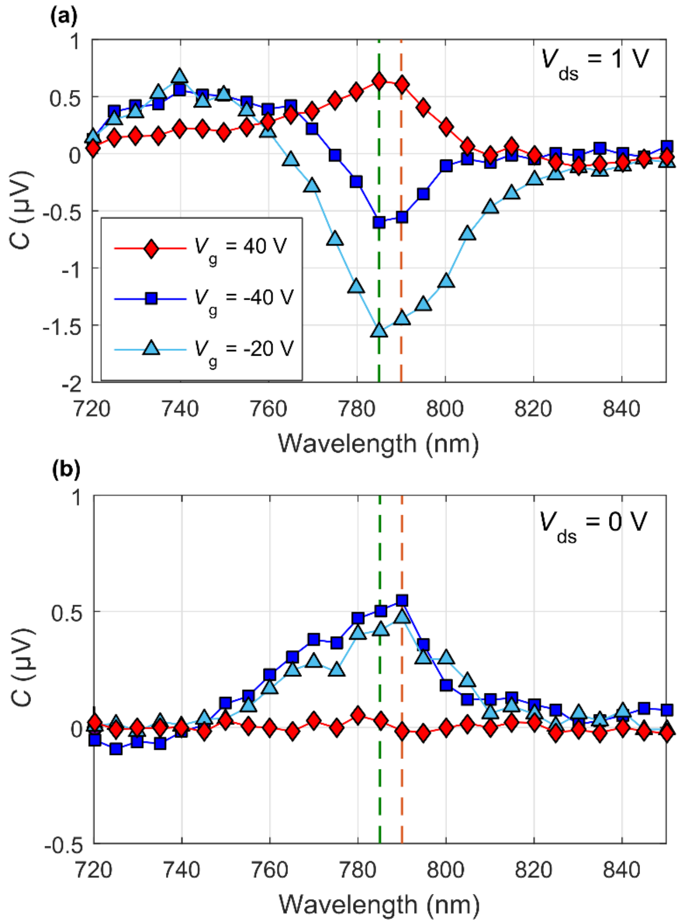

Supplementary Figure 7 Spectral dependence of C at gate voltages, $V_g =$ 40 V, -20 V and -40 V, as labelled, for $V_{ds} = 1$ V (a) and $V_{ds} = 0$ V (b). The spectral measurements corresponding to $V_g = 0$ can be found in the main text (Figure 3c). The dashed vertical lines are guides to the eye for 785 nm (green) and 790 nm (orange).

Supplementary Note 8: Brief note on valley exciton transitions in monolayer $MoSe_2$

The diagram shown in Supplementary Figure 8 summarizes the different excitonic transitions at the band edges of the K valley that can occur for monolayer $MoSe_2$ (similar transitions occur at the K' valley, but all the spins have opposite orientation). For each pair of spin-orbit split subbands one gets an optically active neutral exciton (A^0 and B^0) which, at room temperature, is expected to give absorption peaks at 1.58 eV and 1.78 eV. Positively and negatively charged trion absorption can also occur ($A^{+/-}$ and $B^{+/-}$), at roughly 30 meV lower photon energies. Finally, electrons and holes from subbands with opposite spin can also combine to form the so-called *dark* excitons (A_D^0 and B_D^0). Dark exciton absorption is a priori spin-forbidden, but can become allowed for oblique illumination[15] or even for normal incidence in the presence of a gate voltage[16]. Further review on exciton physics in TMDCs can be found in references [17] and [18].

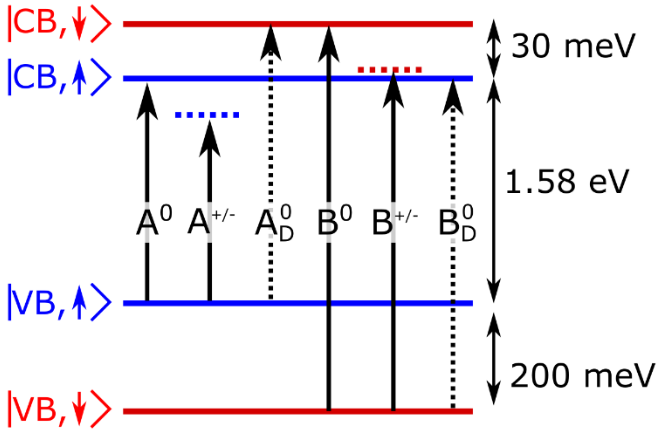

Supplementary Figure 8 Diagram illustrating the possible valley exciton transitions for monolayer $MoSe_2$.

1. Benameur, M. M. *et al.* Visibility of dichalcogenide nanolayers. *Nanotechnology* **22,** 125706 (2011).
2. Golla, D. *et al.* Optical thickness determination of hexagonal Boron Nitride flakes. **161906,** 16–19 (2013).
3. Khestanova, E., Guinea, F., Fumagalli, L., Geim, A. K. & Grigorieva, I. V. Universal shape and pressure inside bubbles appearing in van der Waals heterostructures. *Nat. Commun.* **7,** 1–10 (2016).
4. Baugher, B. W. H., Churchill, H. O. H., Yang, Y. & Jarillo-Herrero, P. Intrinsic electronic transport properties of high-quality monolayer and bilayer MoS2. *Nano Lett.* **13,** 4212–4216 (2013).
5. Ivchenko, E. L. *Optical spectroscopy of semiconductor nanostructures*. (Alpha Science Int'l Ltd., 2005).
6. Belincher, V. I. & Sturman, B. I. The photogalvanic effect in media lacking a center of symmetry. *Physics-Uspekhi* **130,** 415–458 (1980).
7. Sturman, P. J. *Photovoltaic and Photo-refractive Effects in Noncentrosymmetric Materials*. **8,** (CRC Press, 1992).
8. Sipe, J. E. & Shkrebtii, A. I. Second-order optical response in semiconductors. *Phys. Rev. B* **61,** 5337–5352 (2000).
9. Morimoto, T. & Nagaosa, N. Topological nature of nonlinear optical effects in solids. *Sci. Adv.* **2,** e1501524 (2016).
10. De Juan, F., Grushin, A. G., Morimoto, T. & Moore, J. E. Quantized circular photogalvanic effect in Weyl semimetals. *Nat. Commun.* **8,** 15995 (2017).
11. Xiao, D., Liu, G. Bin, Feng, W., Xu, X. & Yao, W. Coupled spin and valley physics in monolayers of MoS2 and other group-VI dichalcogenides. *Phys. Rev. Lett.* **108,** 196802 (2012).
12. Xu, X. Spin and pseudospins in transition metal dichalcogenides. *Proc. - 2014 Summer Top. Meet. Ser. SUM 2014* **10,** 1–2 (2014).
13. Sodemann, I. & Fu, L. Quantum Nonlinear Hall Effect Induced by Berry Curvature Dipole in Time-Reversal Invariant Materials. *Phys. Rev. Lett.* **115,** 1–5 (2015).
14. Golub, L. E. & Ivchenko, E. L. Circular and magnetoinduced photocurrents in Weyl semimetals. (2018). doi:arXiv:1803.02850v1
15. Wang, G. *et al.* In-Plane Propagation of Light in Transition Metal Dichalcogenide Monolayers: Optical Selection Rules. *Phys. Rev. Lett.* **119,** 1–7 (2017).
16. Quereda, J., Ghiasi, T. S., Van Zwol, F. A., Van Der Wal, C. H. & Van Wees, B. J. Observation of bright and dark exciton transitions in monolayer MoSe2 by photocurrent spectroscopy. *2D Mater.* **5,** (2018).
17. Xiao, J., Zhao, M., Wang, Y. & Zhang, X. Excitons in atomically thin 2D semiconductors and their applications. *Nanophotonics* **0,** (2017).
18. Yu, H., Cui, X., Xu, X. & Yao, W. Valley excitons in two-dimensional semiconductors. *Natl. Sci. Rev.* **2,** 57–70 (2015).
